# The preoperative hemoglobin, albumin, lymphocyte, and platelet score (HALP) as a prognostic indicator in patients with non-small cell lung cancer

**DOI:** 10.3389/fnut.2024.1428950

**Published:** 2024-11-28

**Authors:** Qiteng Liu, Hailun Xie, Wenjuan Cheng, Tong Liu, Chenan Liu, Heyang Zhang, Shiqi Lin, Xiaoyue Liu, Haiying Tian, Xiangrui Li, Xin Zheng, Yue Chen, Jinyu Shi, Hong Zhao, Hanping Shi

**Affiliations:** ^1^Department of Gastrointestinal Surgery/Department of Clinical Nutrition, Beijing Shijitan Hospital, Capital Medical University, Beijing, China; ^2^Department of Radiotherapy, Beijing Luhe Hospital, Affiliated to Capital Medical University, Beijing, China; ^3^Beijing International Science and Technology Cooperation Base for Cancer Metabolism and Nutrition, Beijing, China; ^4^Key Laboratory of Cancer FSMP for State Market Regulation, Beijing, China; ^5^Department of Anesthesiology, Beijing Luhe Hospital, Affiliated to Capital Medical University, Beijing, China

**Keywords:** HALP score, NSCLC, cachexia, prognosis, INSCOC study

## Abstract

**Background:**

The Hemoglobin, Albumin, Lymphocyte, and Platelet score (HALP) is an inflammatory nutrition-related biomarker based on hemoglobin and albumin levels, as well as the lymphocyte and platelet load index, which has been reported to be associated with the prognosis of various types of tumors. This study aims to investigate the prognostic value of HALP in patients with non-small cell lung cancer (NSCLC).

**Methods:**

A total of 2,428 patients with NSCLC were enrolled in the INSCOC study. Time-patient survival trends were validated using Kaplan–Meier curves and log-rank tests. The Restricted Cubic Spline function was used to analyze the relationship between the HALP index and overall survival (OS). The Cox regression model was used for univariate and multivariate analyses.

**Results:**

The study included 2,053 patients with detailed biological information and follow-up data, consisting of 1,346 men (65.6%) and 707 women (34.4%). Within this cohort, 138 patients (6.7%) had stage I disease, 282 (13.7%) had stage II, 477 (23.2%) had stage III, and 1,156 (56.3%) had stage IV. A total of 536 patients (26.1%) underwent surgery, 1,494 (72.8%) received chemotherapy, and 271 (13.2%) received radiotherapy. The 1-, 2-, 3-, and 5-year survival rates for patients with NSCLC were 68.6, 48.6, 37.4, and 30.9%, respectively. The optimal cut-off for HALP was determined to be 29.71, with a sensitivity of 53.1% and specificity of 62.9%, leading to the categorization of patients into low (<29.71) (*n* = 963) and high (≥29.71) (*n* = 1,090) HALP groups. Patients with a high HALP demonstrated a significantly higher 5-year overall survival (OS) rate compared to those with a low HALP (38% vs. 23%, *p* < 0.001). Multivariable Cox proportional hazards regression analysis identified that low HALP was an independent risk factor for the survival of patients with non-small cell lung cancer.

**Conclusion:**

The HALP index can be used as an independent prognostic factor for patients with NSCLC, offering clinicians a reference to identify high-risk patient with poor long-term prognoses and improve individualized treatment.

## 1 Introduction

According to the statistics of the global cancer epidemic in 2023, lung cancer remains the tumor with the highest incidence rate and mortality worldwide, with non-small cell lung cancer (NSCLC) being the most common tumor type (1, 2). Currently, although the TNM staging system is regarded as the best criterion for selecting lung cancer treatment and predicting prognosis, patients with the same staging still show significantly different prognoses. Therefore, there is an urgent need to develop simple, cost-effective, and reliable predictive factors in clinical practice to supplement and adjust treatment strategies for prognosis.

Systemic inflammation and nutritional status play important roles in the occurrence, progression, and prognosis of tumors (3–6). The composite index (HALP score) is composed of hemoglobin, albumin, lymphocytes, and platelets, and is considered a new biomarker reflecting systemic inflammation and nutritional status. It has been confirmed as a prognostic factor in several types of cancers (7, 8).

Feng et al. studied 355 patients with Esophageal Squamous Cell Carcinoma (ESCC) who underwent curative resection retrospectively. They found that preoperative HALP had reliable abilities to predict Cancer-Specific Survival (CSS) in respectable ESCC patients in any stage or gender in the subgroup analysis (*p* < 0.001) (9). Xu et al. analyzed 582 pancreatic adenocarcinoma patients who underwent radical resection and discovered that low levels of HALP were significantly associated with lymph node metastasis (10). However, there is limited data on the role of HALP score in the prognosis of non-small cell lung cancer patients, and there is currently a lack of large sample research.

In this study, we assessed the potential of the HALP score in predicting the prognosis of non-small cell lung cancer patients, including the correlation between clinical subgroups and staging outcomes, specifically the length of hospitalization, hospitalization expenses, and cachexia.

## 2 Materials and methods

### 2.1 Study population

This multicenter cohort study utilized data from the INSCOC database (11) (registration number: ChiCTR1800020329).^1^ We employed the design, methodology, and approach described above to prospectively collect cohort data from multiple centers in China. The study involved patients diagnosed with NSCLC between May 2013 and December 2018. All patients included in the INSCOC cohort were 18 years or older, diagnosed with NSCLC, and hospitalized for more than 48 h. These patients also underwent treatments such as surgery, chemotherapy, radiation, and other anticancer therapies. Patients were enrolled at their initial admission, and only data from the first admission were considered for those with multiple hospitalizations. Furthermore, the study excluded patients with immune diseases, insufficient specific data (hemoglobin, albumin, lymphocyte, and platelet levels), and clinical signs of active infection. This study adhered to the principles outlined in the Declaration of Helsinki and received approval from the ethics committee of each local center. Written informed consent was obtained from all participants to utilize their clinical data while maintaining confidentiality regarding personal information.

### 2.2 Data collection

Patient demographics, including sex, age, smoking and drinking history, TNM stage, diabetes, hypertension, coronary disease history, tumor type, and family history of tumors, were extracted from electronic medical records. Overnight fasting venous blood samples were collected within 24 h of admission to measure hemoglobin, albumin, lymphocyte, and platelet levels. These were analyzed and standardized in the central laboratory to eliminate discrepancies due to laboratory equipment. The BMI of all patients was calculated as weight (kg) divided by height (m) squared. The study follows the AJCC Seventh Edition staging system. Patients were categorized into underweight (<18.5 kg/m^2^), normal (18.5–23.9 kg/m^2^), and overweight/ obese (>24 kg/m^2^). The HALP score was defined as: hemoglobin (g/L) × albumin (g/L) × lymphocytes (g/L)/platelets (g/L). The endpoint of this study was OS. OS was defined as the time from admission to death or the last follow-up and measured in months. The final follow-up was conducted on September 1, 2023. Secondary outcomes included 90-day survival and cachexia. The 90-day survival outcome was defined as the patient’s survival status at 90 days after treatment, while cachexia was defined according to the 2011 Delphi international consensus (12).

### 2.3 Statistical analysis

Continuous variables were presented as either mean ± standard deviation or median (interquartile range [IQR]), while categorical variables were presented as numbers and percentages (*n*, %). Independent Student’s t-test or nonparametric test was used for comparing continuous variables, and the chi-squared test or Fisher’s exact test was used for comparing categorical variables. The Receiver Operating Characteristic (ROC) curve was utilized to determine the optimal cut-off value for HALP. Time-patient survival trends were analyzed using Kaplan–Meier curves and log-rank tests. Univariate and multivariate analyses were conducted using the Cox regression model. Only significant factors from the univariate COX analysis (*p* < 0.05) were included in the multivariate analysis. Sensitivity analysis excluded patients who passed away within 6 months. A two-sided *p*-value <0.05 was considered statistically significant. All statistical analyses were carried out using R software version 4.1.1.

## 3 Results

### 3.1 Demographic and clinicopathological features of NSCLC patients

**Figure 1:**
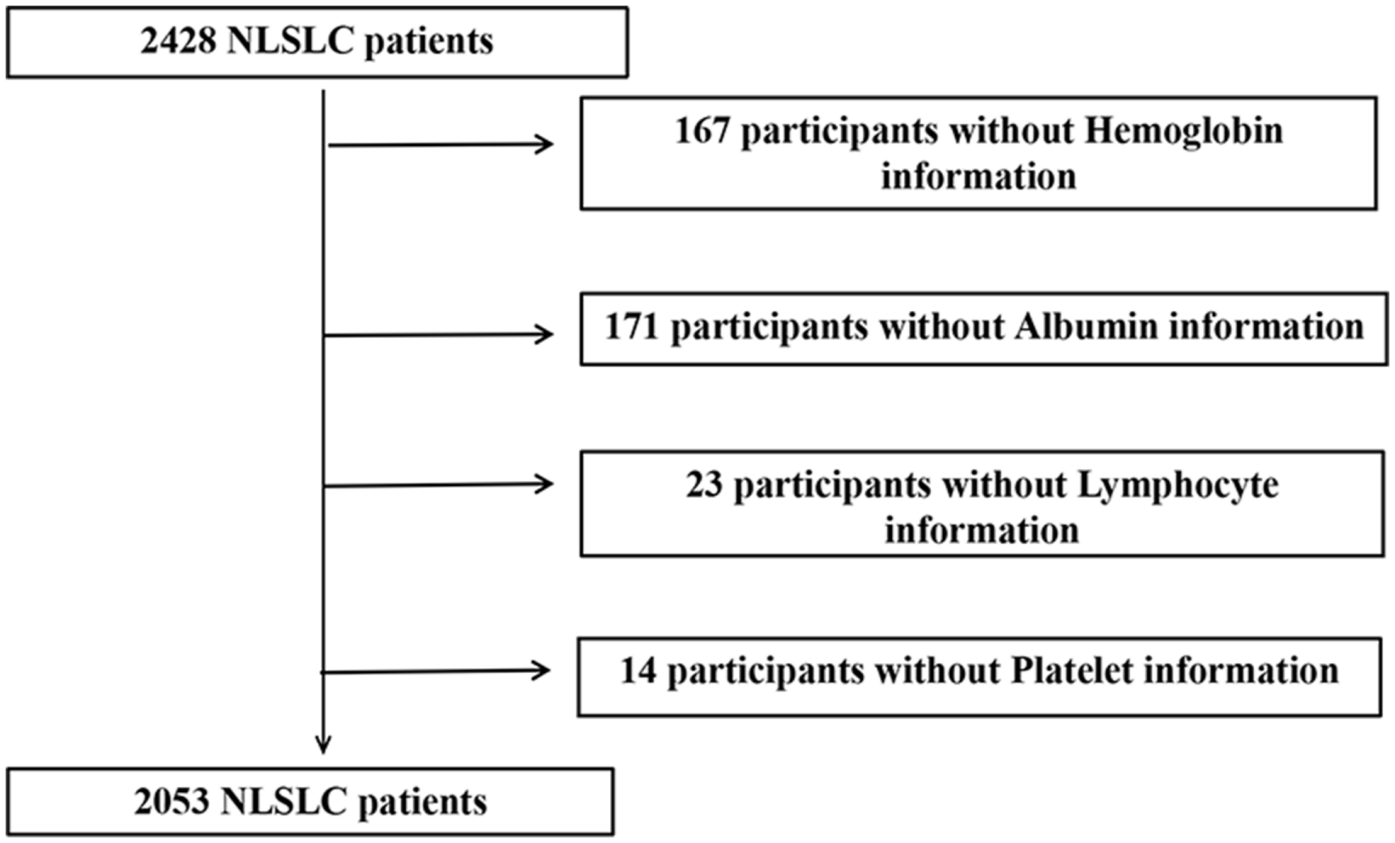
A flowchart of the screening process.

Initially, 2,428 patients with NSCLC were enrolled in the INSCOC study. After excluding patients with missing serological data, 2,053 patients with NSCLC were included in the study, comprising 1,346 men (65.6%) and 707 women (34.4%). A flowchart of the screening process is shown in Figure 1. The mean age of the participants was 60.73 (±9.8) years. In this cohort, 138 patients (6.7%) had stage I disease, 282 (13.7%) had stage II disease, 477 (23.2%) had stage III disease, and 1,156 (56.3%) had stage IV disease. A total of 536 (26.1%) patients underwent surgery. Notably, there were 1,243 (60.5%) smokers, 529 (25.8%) drinkers, 1,494 (72.8%) who underwent chemotherapy, 271 (13.2%) patients who received radiotherapy, 209 (10.1%) with diabetes, and 433 (21%) with hypertension. The demographic and clinicopathological features of the patients are summarized in Table 1.

**Table 1:** The clinicopathological features in patients with lung cancer.

Characteristic	Overall *I* = 2,053
Sex, men, *n* (%)	1,346 (65.6)
Sex, women, *n* (%)	707 (34.4)
Age, years, mean (SD)	60.73 (9.80)
BMI (median [IQR])	22.67 (20.57, 24.89)
Hypertension, yes, *n* (%)	431 (21)
Diabetes, yes, *n* (%)	209 (10.2)
Smoking yes, *n* (%)	1,243 (60.5)
Drinking, yes, *n* (%)	529 (25.8)
Family history, yes, *n* (%)	373 (18.2)
TNM stage, *n* (%)	
Stage I	138 (6.7)
Stage II	282 (13.7)
Stage III	477 (23.2)
Stage IV	1,156 (56.3)
Surgery, yes, *n* (%)	536 (26.1)
Radiotherapy, yes, *n* (%)	271 (13.2)
Chemotherapy, yes, *n* (%)	1,494 (72.8)
White blood cells (median [IQR])	6.46 (5.10, 8.28)
Neutrophil (median [IQR])	4.20 (2.95, 5.85)
Lymphocyte (median [IQR])	1.50 (1.10, 1.93)
Platelets (median [IQR])	236.00 (182.00, 297.00)
Red blood cells (median [IQR])	4.33 (3.91, 4.72)
Hemoglobin (median [IQR])	130 (116, 141)
Albumin (median [IQR])	39.20 (35.90, 42.00)
CRP (median [IQR])	5.81 (2.96, 22.83)
KPS score (median [IQR])	90.00 (80.00, 90.00)
PGSGA score (median [IQR])	4.00 (2.00, 8.00)
Cachexia, yes, *n* (%)	553 (27.2)
NRS2002 (median [IQR])	1.00 (0.00, 2.00)
Global quality of life score (median [IQR])	48.00 (44.00, 56.00)
Short-term outcome, yes, *n* (%)	167 (8.1)
Status, death, *n* (%)	1,135 (55)
Length of hospitalization (median [IQR])	10.00 (7.00, 15.00)
Hospitalization expenses (median [IQR])	14,784.75 (9,509.52, 23,451.30)

CRP, C-reactive protein; Hb, hemoglobin; BMI, body mass index.

### 3.2 Comparison of the clinicopathological characteristics between low and high HALP

**Figure 2:**
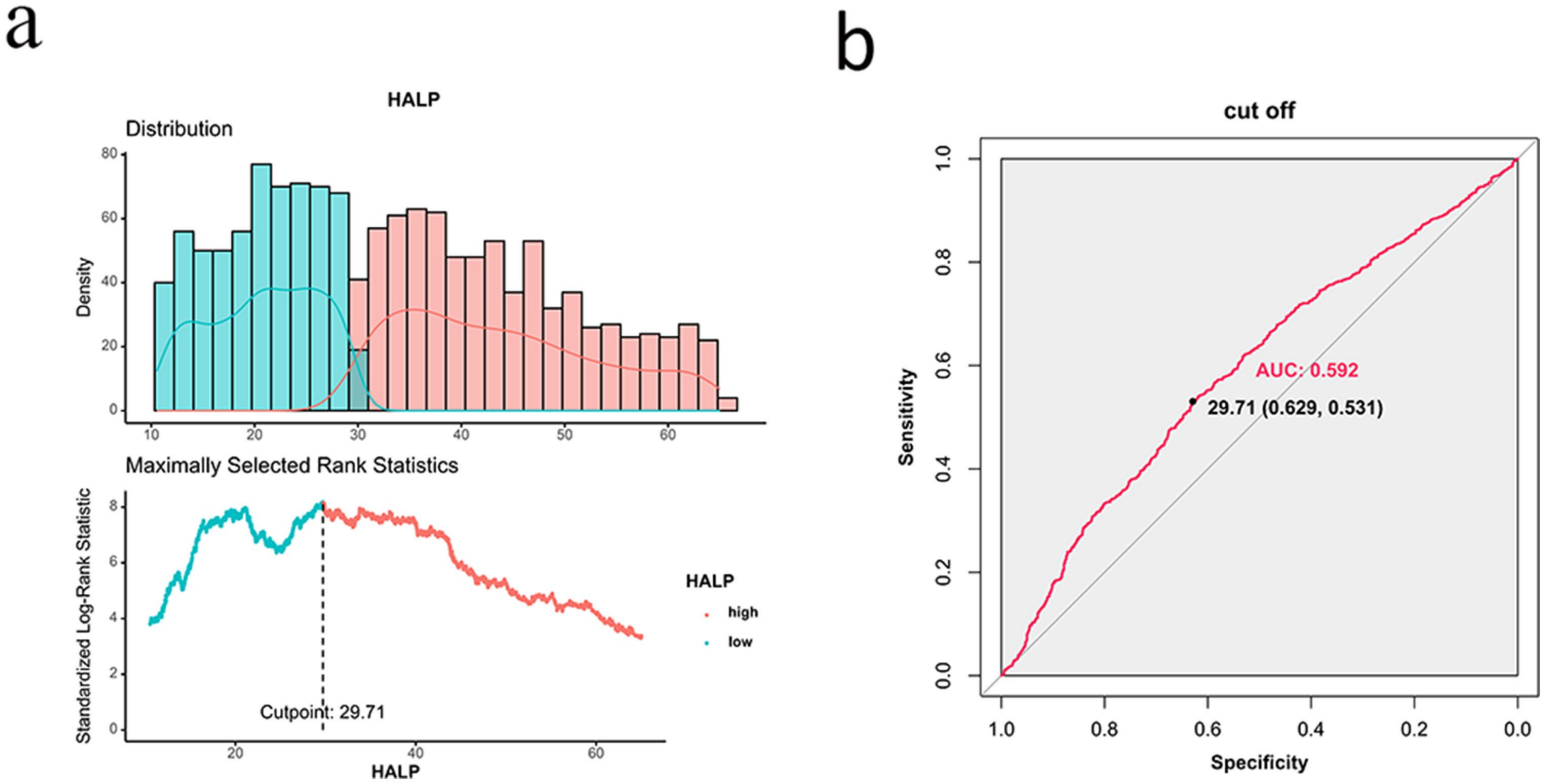
ROC curve of HALP for predicting survival outcome of patients with NSCLC. **(A)** Distribution of HALP scores and determination of the optimal cutoff point (29.71). **(B)** ROC curve of HALP, showing an AUC of 0.592.

The ROC curve for HALP predicting survival was plotted using patient survival as the outcome variable. The area under the ROC curve was 0.592 with a 95% CI of 0.531–0.629. The optimal cut-off value was determined to be 29.17, with a sensitivity of 53.1% and a specificity of 62.9% (Figure 2). The patients were divided into a low HALP group (*n* = 963) and a high HALP group (*n* = 1,090). There were no significant differences in age, sex, hypertension, diabetes, drinking habits, family history of tumors, and chemotherapy between the groups (*p* > 0.05). However, there were notable variances in BMI, smoking history, TNM stage, radiotherapy, and surgery between the two groups (*p* < 0.05) as shown in Table 2.

**Table 2:** Characteristics by level of HALP index in patients with lung cancer.

Characteristic	HALP		
	Low *n* = 963	High *n* = 1,090	*p* value
Sex, Male, *n* (%)	615 (63.9)	731 (67.1)	<0.14
Sex, Female, *n* (%)	348 (36.1)	359 (32.9)	
Age, years, mean (SD)	60.73 (10.03)	60.72 (9.60)	<0.999
BMI (median [IQR])	22.07 (20.00, 24.43)	23.22 (21.15, 25.30)	<0.001
Hypertension, yes, *n* (%)	221 (22.9)	210 (19.3)	0.047
Diabetes, yes, *n* (%)	92 (9.6)	117 (10.7)	0.418
Smoking yes, *n* (%)	547 (56.8)	696 (63.9)	<0.001
Drinking, yes, *n* (%)	240 (24.9)	289 (26.5)	0.44
Family history, yes, *n* (%)	168 (17.4)	205 (18.8)	0.459
TNM stage, *n* (%)			<0.001
Stage I	34 (3.5)	104 (9.5)	
Stage II	102 (10.6)	180 (16.5)	
Stage III	220 (22.8)	257 (23.6)	
Stage IV	607 (63.0)	549 (50.4)	
Surgery, yes, *n* (%)	196 (20.4)	340 (31.2)	<0.001
Radiotherapy, yes, *n* (%)	162 (16.8)	109 (10.0)	<0.001
Chemotherapy, yes, *n* (%)	688 (71.4)	806 (73.9)	0.222
White blood cells (median [IQR])	6.61 (5.15, 8.66)	6.35 (5.04, 7.97)	<0.015
Neutrophil (median [IQR])	4.55 (3.05, 6.45)	3.90 (2.87, 5.33)	<0.001
Lymphocyte (median [IQR])	1.17 (0.84, 1.51)	1.80 (1.43, 2.19)	<0.001
Platelets (median [IQR])	281 (218, 345)	206 (164, 252)	<0.001
Red blood cells (median [IQR])	4.21 (3.72, 4.58)	4.44 (4.07, 4.84)	<0.001
Hemoglobin (mean (SD))	117.22 (30.3)	133.69 (17.88)	<0.001
Albumin (median [IQR])	37.30 (33.80, 40.50)	40.50 (37.80, 43.00)	<0.001
PGSGA score (median [IQR])	5.00 (2.00, 9.00)	3.00 (2.00, 6.00)	<0.001
NRS2002 (median [IQR])	1.00 (1.00, 3.00)	1.00 (0.00, 2.00)	<0.001
Global quality of life score (median [IQR])	50.00 (45.00, 58.00)	47.00 (43.00, 53.00)	<0.001
KPS score (median [IQR])	90.00 (80.00, 90.00)	90.00 (80.00, 90.00)	<0.001
90-day outcomes, yes, *n* (%)	125 (13.0)	42 (3.9)	
Cachexia, yes, *n* (%)	319 (33.5)	234 (21.6)	<0.001
Status, death, *n* (%)	352 (36.6)	570 (52.3)	<0.001
Length of hospitalization (median [IQR])	611 (63.4)	520 (47.7)	0.308
Hospitalization expenses (median [IQR])	10.00 (7.00, 16.00)	10.00 (7.00, 15.00)	0.016

### 3.3 The Kaplan–Meier survival analysis of the HALP score for patients with NSCLC

**Figure 3:**
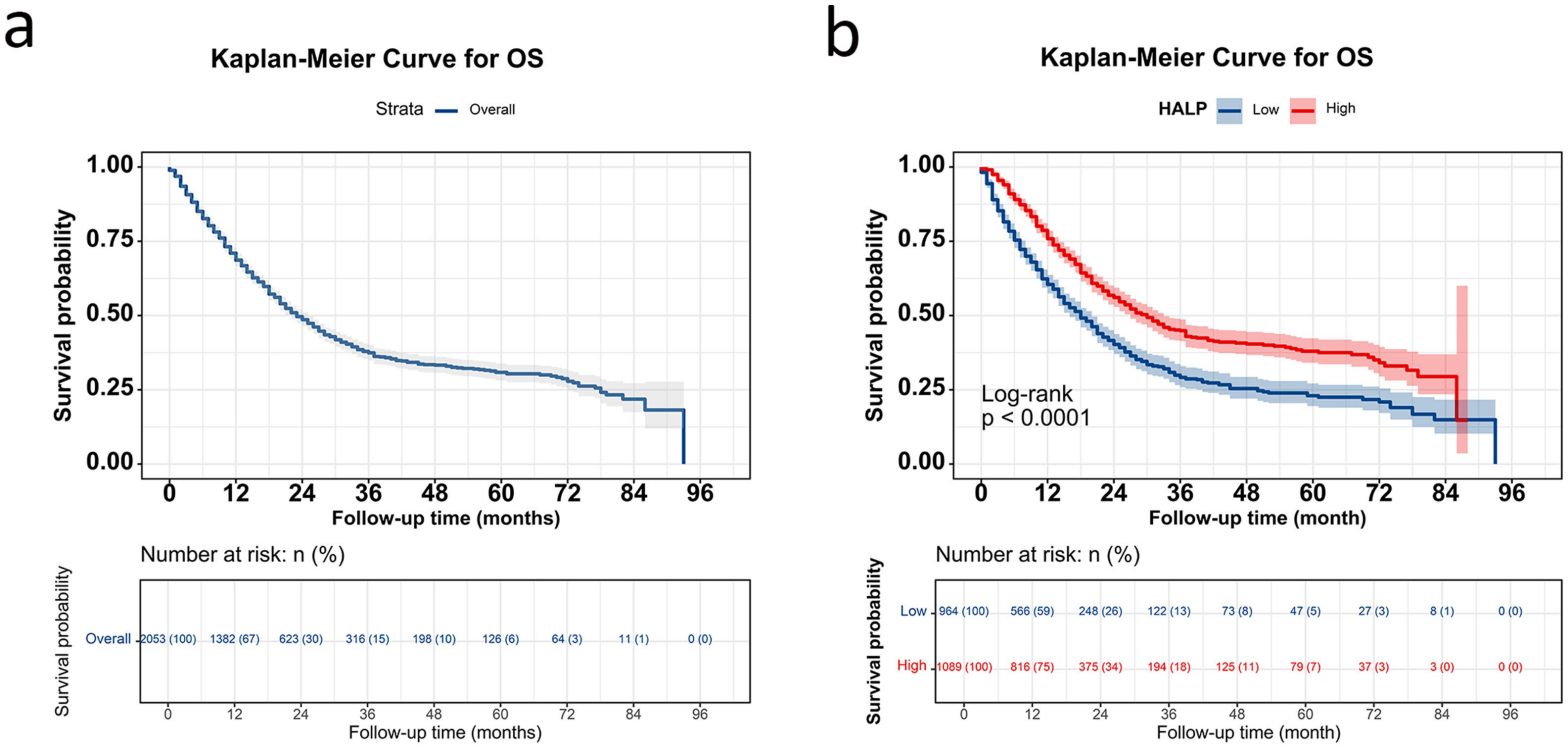
Kaplan–Meier curve in patients with NSCLC. **(A)** Overall survival curve for all patients; **(B)** survival curve grouped by HALP index.

**Figure 4:**
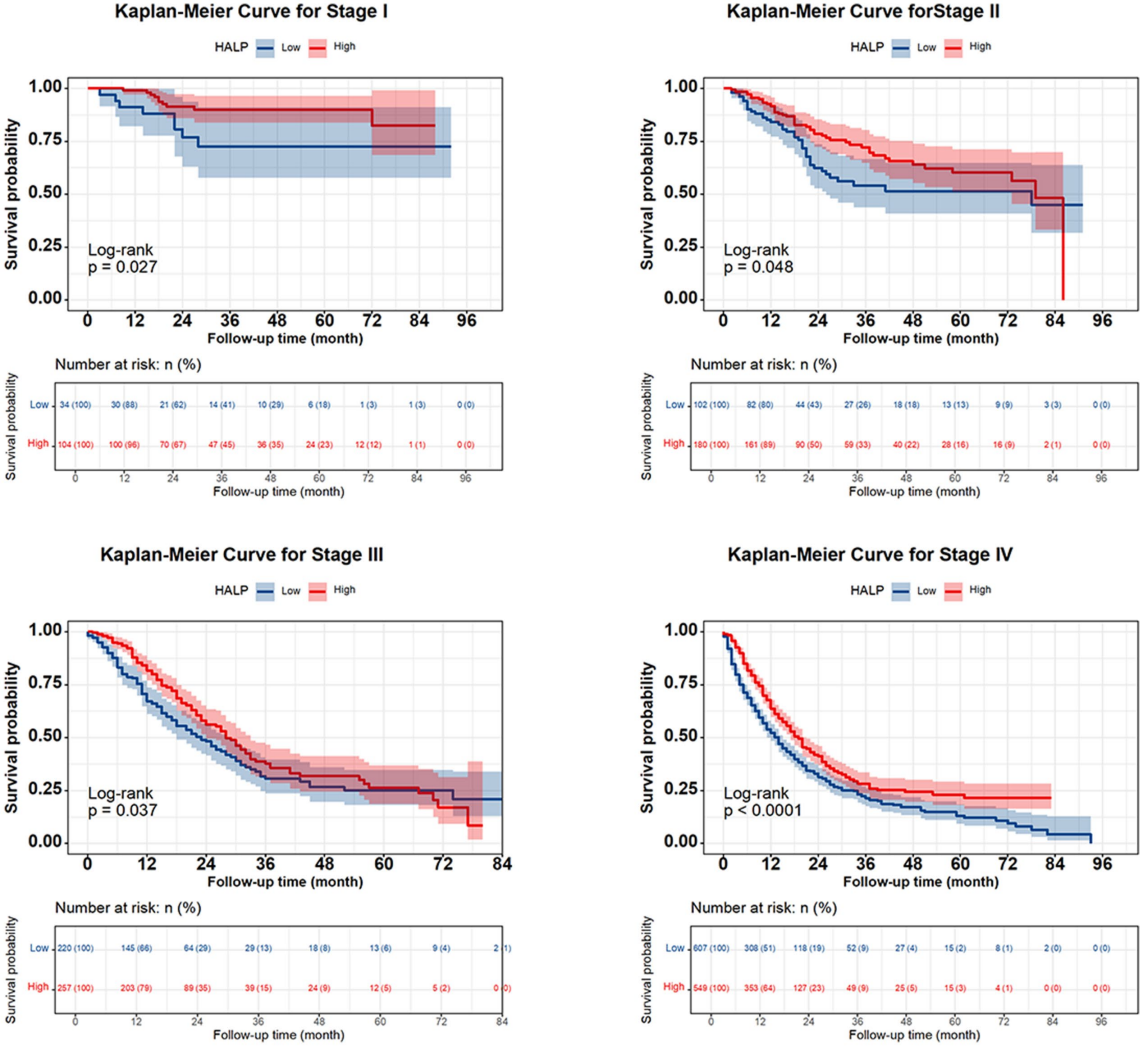
Kaplan–Meier curve of the HALP index in patients with all stages of NSCLC.

**Figure 5:**
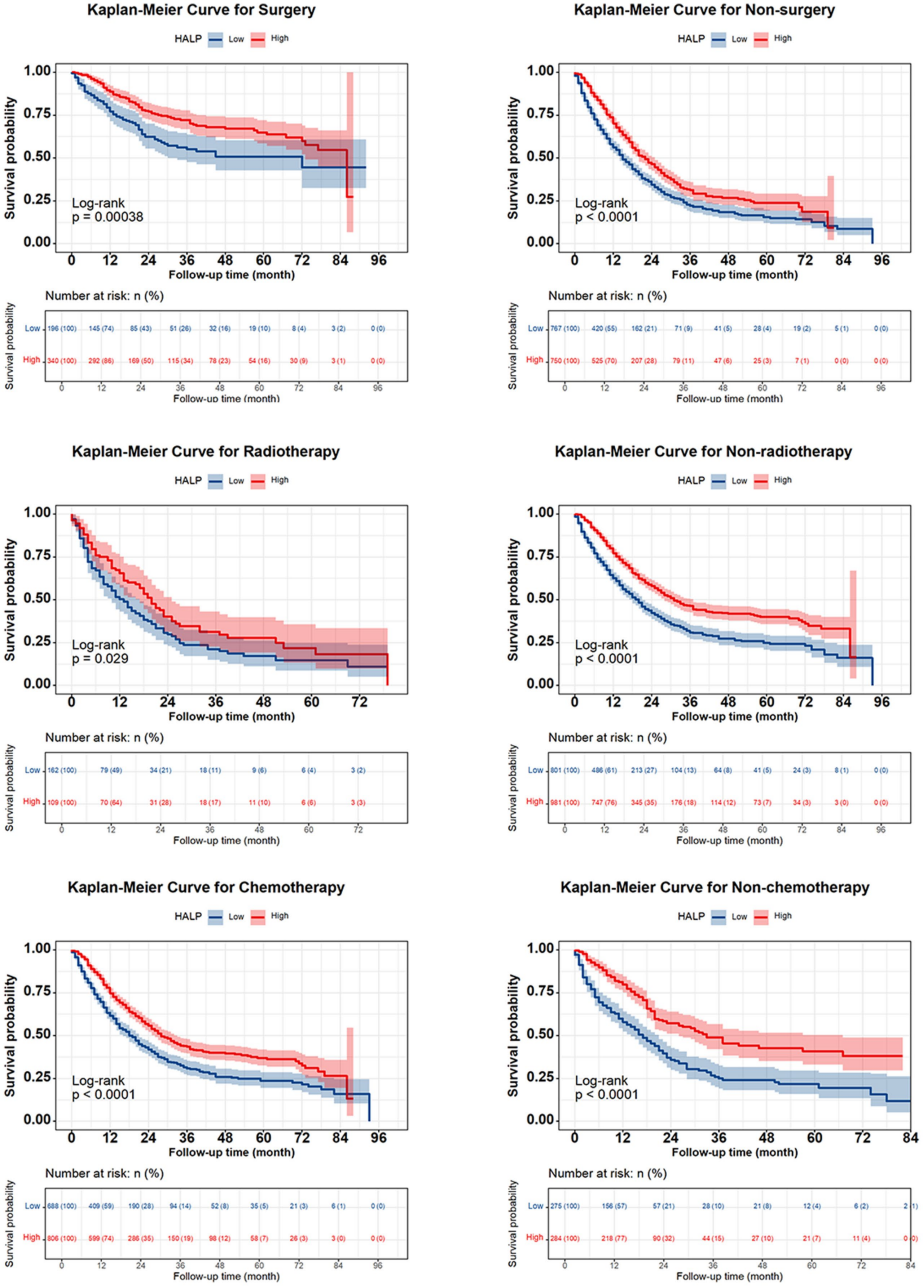
Kaplan Meier curve of HALP index in NSCLC subgroups of patients undergoing chemotherapy, radiotherapy, and surgery.

The 1-, 2-, 3-, and 5-year survival rates for patients with NSCLC were 68.6, 48.6, 37.4, and 30.9%, respectively. Patients with high HALP demonstrated significantly better outcomes than those with low HALP (*p* < 0.001) (Figure 3). The 5-year survival rate in the higher HALP group was 38%, which was higher than that in the lower HALP group (23%). Notably, regardless of the stage, the overall survival (OS) in the high HALP group was better than that of the low HALP group (Figure 4). In patients at all stages, high HALP represents a better prognosis, especially as the staging advances, the more significant the statistical difference. Additionally, it was found that HALP can effectively predict the prognosis of subgroups of patients receiving and not receiving chemotherapy, surgery, and radiation therapy (Figure 5).

### 3.4 HALP for predicting survival outcomes of patients with NSCLC

**Figure 6:**
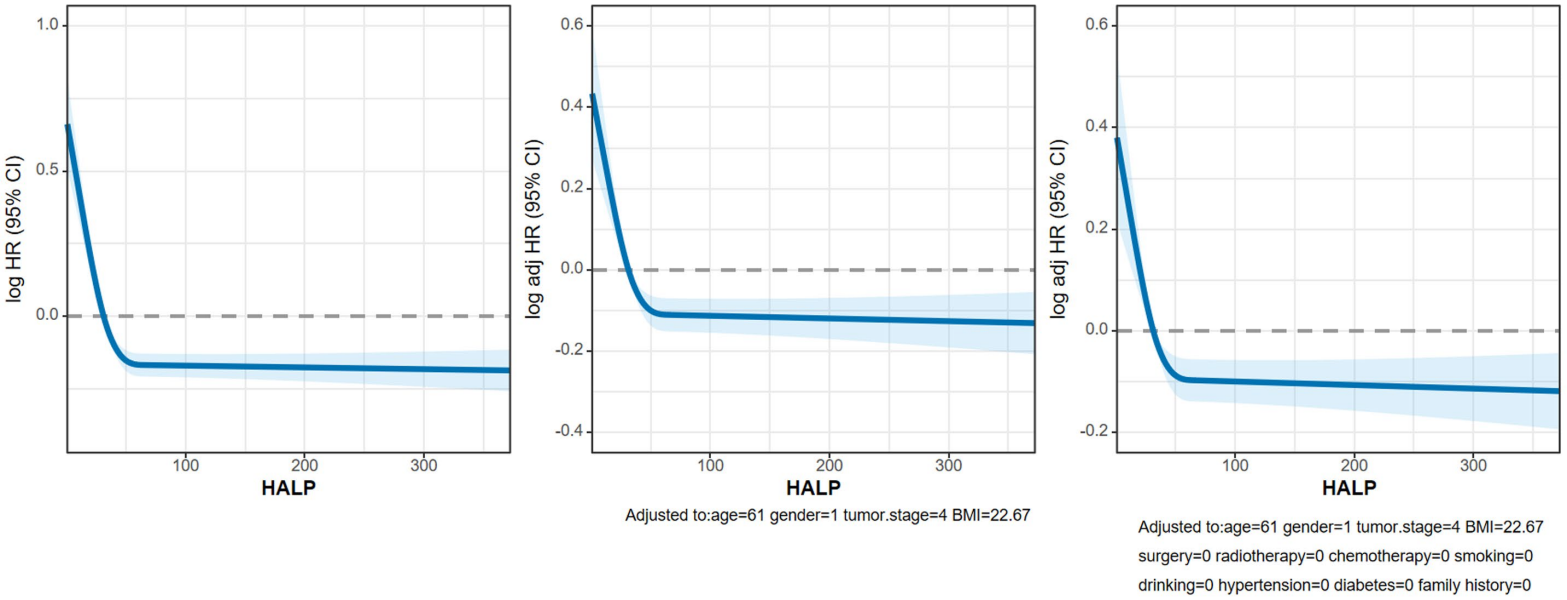
The RCS curves of association between HALP and OS in patients with NSCLC. Model a: Not adjusted. Model b: Adjusted for age, sex, BMI and TNM stage. Model c: Adjusted for age, sex, BMI, TNM stage, surgery, radiotherapy, chemotherapy, hypertension, diabetes, smoking, drinking and family history.

We conducted an analysis on the association between the HALP score and overall survival (OS) in patients with NSCLC using multivariate-adjusted restricted cubic spline (RCS) with three knots. Our findings revealed an inverted L-shaped dose–response relationship when analyzed as a continuous variable (Figure 6), indicating that patients with lower HALP levels tended to have poorer prognoses. Furthermore, the multivariable Cox proportional hazards regression analysis showed that low HALP was an independent risk factor for NSCLC prognosis (hazard ratio [HR] = 0.756, 95% CI: 0.671–0.853, *p* < 0.001). Compared to the Q1 group used as a reference, the risk of adverse prognosis in the Q2, Q3, and Q4 groups progressively decreased, with hazard ratios (HRs) of 0.754, 0.697, and 0.662, respectively (Table 3).

**Table 3:** Association between HALP and overall survival of patients with lung cancer.

HALP	Model a	*p* value	Model b	*p* value	Model c	*p* value
Continuous (per SD)	0.77 (0.493, 1.2)	0.248	0.849 (0.552, 1.306)	0.456	0.842 (0.548, 1.295)	0.434
Cutoff value		<0.001		<0.001		<0.001
C1 (<29.7)	ref		ref		ref	
C2 (≥29.7)	0.613 (0.546, 0.69)		0.725 (0.644, 0.817)		0.756 (0.671, 0.853)	
Quartiles						
Q1 (<19.9)	ref		ref		ref	
Q2 (19.9–31.3)	0.718 (0.614, 0.838)	<0.001	0.743 (0.636, 0.869)	<0.001	0.754 (0.645, 0.881)	<0.001
Q3 (31.3–46.5)	0.562 (0.478, 0.662)	<0.001	0.666 (0.565, 0.784)	<0.001	0.697 (0.591, 0.823)	<0.001
Q4 (>46.5)	0.504 (0.427, 0.595)	<0.001	0.626 (0.53, 0.74)	<0.001	0.662 (0.559, 0.784)	<0.001
*p* for trend	0.79 (0.749, 0.833)	<0.001	0.854 (0.809, 0.902)	<0.001	0.872 (0.825, 0.921)	<0.001

Model a: No adjusted.

Model b: Adjusted for age, sex, BMI, TNM stage.

Model c: Adjusted for age, sex, BMI, TNM stage, surgery, radiotherapy, chemotherapy, hypertension, diabetes, smoking, drinking, family history.

### 3.5 Logistic regression analysis of the HALP and secondary outcomes

**Figure 7:**
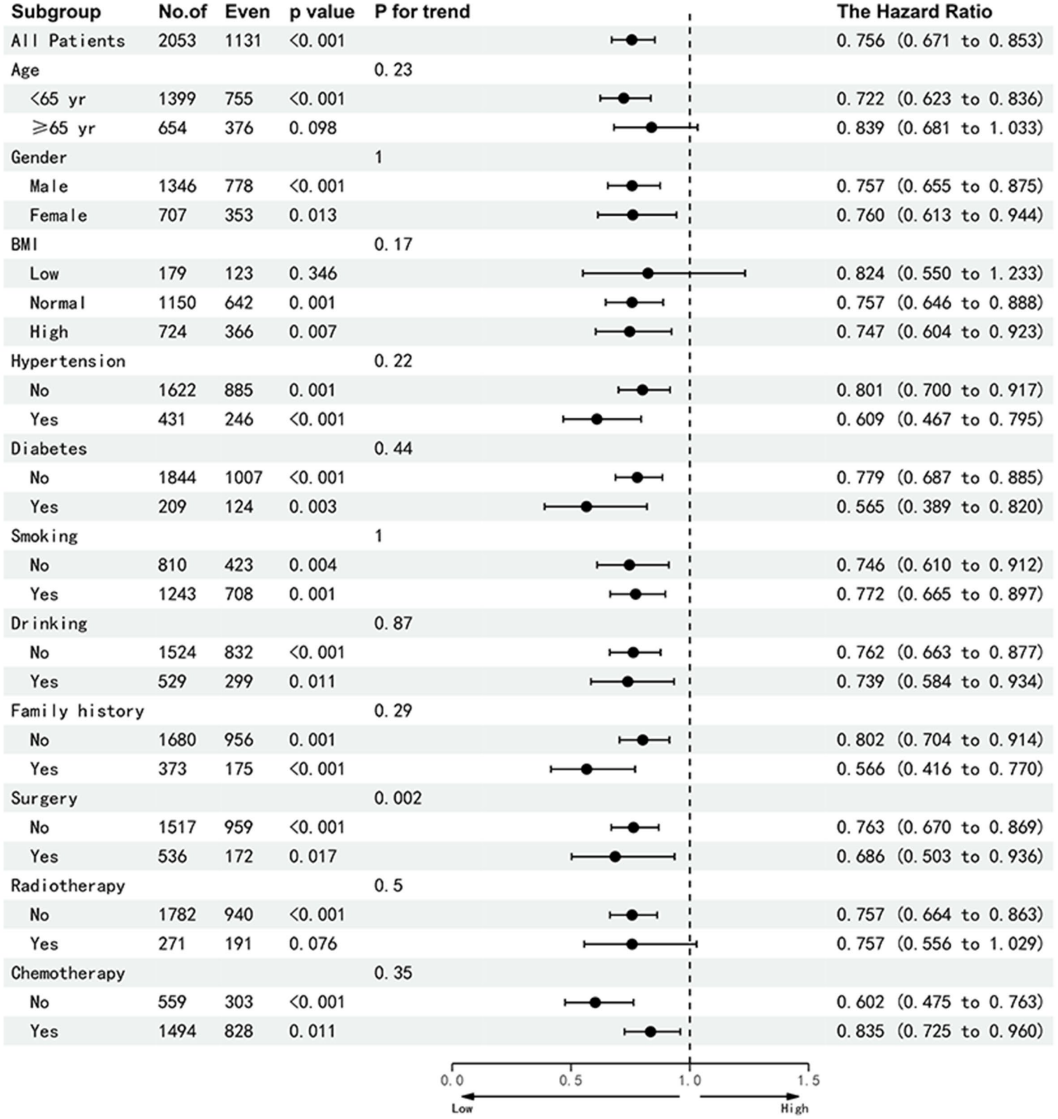
Sub-group analysis of the HALP index in patients with NSCLC. Adjusted for age, gender, tumor stage, BMI, smoking, alcohol drinking, radiotherapy, chemotherapy, surgery, diabetes, hypertension and family history of tumors. Abbreviations: BMI, body mass index; HR, hazard rati.

In this study, 167 patients (8.1%) experienced adverse outcomes within 90 days. Multivariable-adjusted logistic regression analysis identified low HALP as an independent risk factor for adverse 90-day outcomes in patients with NSCLC (OR = 0.35, 95% CI: 0.24–0.51; *p* < 0.001). Furthermore, 553 patients (27.2%) with NSCLC were diagnosed with cachexia. Essentially, HALP was also an independent factor affecting cachexia (OR = 0.76, 95% CI: 0.61–0.995, *p* < 0.001). In comparison with the Q1 group, the odds ratios for the Q2, Q3, and Q4 groups were 0.86, 0.87, and 0.60, respectively (Table 4). Patients were grouped according to age, sex, TNM stage, BMI, smoking, alcohol consumption, chemotherapy, radiation, surgery, diabetes, hypertension, and family history of tumors. Subsequent multivariable subgroup analysis revealed that a low HALP score is an independent risk factor across most subgroups, except for the radiotherapy group, the low BMI, Stage II and III subgroup (Figure 7). Among all patients, a low HALP score was significantly associated with an increased risk of mortality (HR = 0.756, 95% CI: 0.671–0.85, *p* < 0.001). In the age subgroup, low HALP scores were significantly linked to poorer prognosis in patients under 65 years (HR = 0.722, 95% CI: 0.623–0.83, *p* < 0.001), whereas no significant association was observed in patients aged 65 and older (HR = 0.839, 95% CI: 0.681–1.03, *p* = 0.098). For the low BMI group, the HR for a low HALP score was 0.824 (95% CI: 0.550–1.233, *p* = 0.346), showing no significant difference. In the staging subgroup, low HALP scores were significantly associated with prognosis in stage I (HR = 0.271, 95% CI: 0.086–0.849, *p* = 0.025) and stage IV (HR = 0.748, 95% CI: 0.646–0.845, *p* < 0.001) patients, but showed no significant effect in stage II and III patients (*p* = 0.103 and 0.137, respectively).

**Table 4:** Logistic regression analysis of HALP associated with secondary outcomes.

HALP	Model a	*p* value	Model b	*p* value	Model c	*p* value
90-day outcomes						
Continuous (per SD)	0.37 (0.02, 6.41)	0.492	0.59 (0.05, 6.75)	0.675	0.59 (0.05, 6.75)	0.675
Cutoff value		<0.001		<0.001		<0.001
C1 (<29.7)	ref		ref		ref	
C2 (≥29.7)	0.27 (0.19, 0.39)	<0.001	0.34 (0.23, 0.49)	<0.001	0.35 (0.24, 0.51)	<0.001
Quartiles						
Q1 (<19.9)	ref		ref		ref	
Q2 (19.9–31.3)	0.309 (0.203, 0.468)	<0.001	0.324 (0.211, 0.499)	<0.001	0.334 (0.215, 0.519)	<0.001
Q3 (31.3–46.5)	0.229 (0.145, 0.363)	<0.001	0.281 (0.175, 0.453)	<0.001	0.293 (0.180, 0.477)	<0.001
Q4 (>46.5)	0.135 (0.077, 0.236)	<0.001	0.183 (0.103, 0.325)	<0.001	0.196 (0.109, 0.352)	<0.001
Cachexia						
Continuous (per SD)	0.56 (0.17, 1.82)	0.332	0.66 (0.21, 2.03)	0.465	0.69 (0.23, 2.05)	0.507
Cutoff value		<0.001		<0.001		<0.001
C1 (<29.7)	ref		ref		ref	
C2 (≥29.7)	0.55 (0.45, 0.67)	<0.001	0.75 (0.61, 0.93)	0.01	0.76 (0.61, 0.95)	0.016
Quartiles						
Q1 (<19.9)	ref		ref		ref	
Q2 (19.9–31.3)	0.738 (0.567, 0.96)	0.02	0.84 (0.63, 1.15)	0.2	0.86 (0.65, 1.15)	0.31
Q3 (31.3–46.5)	0.598 (0.457, 0.783)	<0.001	0.86 (0.64, 1.15)	0.29	0.87 (0.65, 1.18)	0.39
Q4 (>46.5)	0.387 (0.29, 0.518)	<0.001	0.58 (0.42, 0.79)	<0.001	0.60 (0.43, 0.82)	<0.001

Model a: No adjusted.

Model b: Adjusted for age, sex, BMI, TNM stage.

Model c: Adjusted for age, sex, BMI, TNM stage, surgery, radiotherapy, chemotherapy, hypertension, diabetes, smoking, drinking, family history.

## 4 Discussion

TNM staging is currently the most commonly used prognostic indicator for tumors. However, even at the same stage, the prognosis of patients can still vary, so it is necessary to develop an affordable and convenient prognostic factor to assist in predicting outcomes.

As is well known, cancer is a chronic debilitating disease, and the hemoglobin levels of patients are associated with survival rate and tumor progression (13, 14). Ferran-Carpintero et al. reported that preoperative anemia was common in patients undergoing radical cystectomy for bladder cancer and is related to a worse cancer prognosis, making it a modifiable preoperative factor (15). Serum albumin can be used as an indicator of nutritional status to assess the survival rate of cancer patients (16). Wang et al. reported that patients with hypoalbuminemia and anemia are more likely to have higher tumor staging in early cervical cancer, leading to a worse prognosis than those without these conditions (17). Studies have indicated that the inflammatory microenvironment and infiltrating immune system cells are key components of tumor formation (18). Lymphocytes and platelets are fundamental components of systemic inflammation, representing the sustained inflammatory microenvironment (19, 20). For example, lymphocytes can release a series of cytokines, inhibit tumor cell growth, and are crucial for anti-tumor immunity (21). Lymphocyte depletion can predict the survival of advanced tumors (22). Platelets release cytokines, promote tumor growth, invasion, and angiogenesis, and play a role in regulating the tumor microenvironment (23). The routine blood test based on the platelet to lymphocyte ratio (PLR) is a potential biomarker for systemic inflammatory response and can act as a prognostic marker for the survival of various malignant tumors (24). Previous studies, mainly from East Asia, have shown that a high PLR is associated with a poor prognosis in several tumor types (25).

Albumin and hemoglobin levels, as well as lymphocyte and platelet counts, are common clinical biomarkers that constitute the HALP score, first introduced in 2015 by Chen et al. for predicting survival outcomes in patients with gastric cancer (8). However, their utility in predicting the prognosis of non-small cell lung cancer (NSCLC) is unclear.

Several small-sample studies have explored the prognostic relationship between HALP and NSCLC. Güç et al. (26) included 401 patients with NSCLC. The results showed that the cut-off points were found to be 23.24 (AUC = 0.928; 95% CI: 0.901–0.955, *p* < 0.001), and multivariate analysis revealed that HALP groups with lower HALP scores had significantly shorter overall survival compared to those with higher HALP scores (HR = 2.988, 95% CI: 2.065–4.324, *p* < 0.001), which is consistent with our findings. Our study with 2,053 patients demonstrated that the HALP score was an independent predictive factor, effectively predicting the overall survival of NSCLC patients (HR = 0.756, 95% CI: 0.671–0.853, *p* < 0.001). The optimal cut-off for HALP was 29.71, and patients with higher HALP scores exhibited significantly better outcomes than those with low HALP scores. For patients at all stages, a high HALP score indicates a better prognosis, with the statistical difference becoming more significant as the staging advances. Furthermore, we observed that HALP can effectively predict the prognosis of subgroups of patients undergoing various treatments, including chemotherapy, surgery, and radiation therapy. This suggests that the HALP score can predict the prognosis of NSCLC across different stages and treatment modalities.

Although this study provides evidence supporting the prognostic significance of the HALP score in lung cancer, the specific mechanism linking a low HALP score to decreased survival rates and unfavorable clinical outcomes remains uncertain. There were significant differences in BMI, smoking history, TNM stage, radiotherapy, surgery, and hematological indicators between the HALP groups in our study. Zhang et al. reported that BMI and TNM stage are independent risk factors that affect the prognosis of patients with lung cancer (27). In this study, the later the staging of patients, the lower the HALP, indicating a poorer prognosis. At the same time, the operation rate of the high HALP group is also higher, which means that patients who can undergo surgery tend to have a better prognosis. Additionally, we observed a notable interaction between the HALP score and surgery, indicating their combined effects on patient outcomes.

Regarding hematological indicators, a higher HALP score represents higher hemoglobin, albumin, lymphocyte, and lower platelet levels. Xu et al. found that a decrease in serum lymphocyte and albumin levels may lead to a poor prognosis in patients undergoing pancreatic cancer resection (10). Anemia is a common clinical feature in cancer patients and might contribute to hypoxia (28), which could drive cancer progression and therapeutic resistance and is closely related to poor survival (29). Al-Shaiba reported that hypoalbuminemia is associated with the presence of a systemic inflammatory response in patients with colorectal liver metastases and impairs host immunity, contributing to poor oncologic outcomes (30). In addition, lymphocytes are crucial in cancer immune surveillance by promoting tumor cell apoptosis and infiltrated lymphocytes (CD4+/CD8+ T-lymphocytes), which are among the most important members of host immunity (31).

Serum platelet levels reflect systemic inflammatory responses as the basis of the HALP score. The inflammatory state is a crucial prognostic indicator of liver metastasis in colorectal cancer (32). We observed that the HALP score is influenced by changes in platelet levels, suggesting that patients with lower platelet counts tend to have a better prognosis. Platelets play a role in the development and progression of cancer by regulating the tumor microenvironment. They release a wide range of proteins, including growth and angiogenic factors, lipids, and extracellular vesicles rich in genetic material. These substances can induce phenotypic changes in target cells, such as immune, stromal, and tumor cells, promoting carcinogenesis and the formation of metastases (33). Importantly, tumor cells can evade recognition by the immune system by activating platelets and binding with them to form tumor thrombi (34).

The 90-day mortality rate is a criterion used to evaluate surgical interventions and improve cancer treatment plans. The prognosis and treatment strategies are better for patients with a low 90-day mortality rate. In this study, patients with a high HALP not only experience a survival benefit but also exhibit decreased 90-day mortality and cachexia (35). Cachexia, which occurs in the late stages in most patients, is a multifactorial syndrome characterized by progressive loss of skeletal muscle mass, along with adipose tissue wasting, systemic inflammation, and other metabolic abnormalities leading to functional impairment, and represents a poor prognosis (36). In previous studies, the prevalence of tumor cachexia in China was reported to be as high as 37% (12). In this study, the incidence of cachexia was 27%, which may be influenced by factors such as region, ethnicity, and diagnostic criteria. Our findings suggest that the HALP indicator could serve as a predictive tool for the early identification of cachexia and 90-day mortality, aiding in the selection of multimodal treatment strategies in clinical practice.

In the multivariate analysis, the HALP score serves as a protective factor in patients with lung cancer. However, the high HALP group did not show significant survival benefits in the sub-analysis of radiotherapy and lower BMI. This may be attributed to the fact that patients with lower BMI are more likely to receive adequate nutritional guidance. Furthermore, given the clinical response to radiotherapy, radiotherapy physicians emphasize nutritional support during the treatment, which could impact the HALP score in this subgroup. Of course, the sample size of the radiotherapy group and data bias are also inevitable factors influencing the outcomes.

Our findings indicate that the HALP score independently functions as a prognostic factor for overall survival (OS) in patients diagnosed with NSCLC. As a novel marker of inflammation and nutrition, the HALP score is an improved immune nutrition scoring system that is inexpensive, easily accessible, and requires simple calculations. For patients with a low HALP score and poorer prognosis, physicians may adopt a more aggressive treatment approach, such as intensified chemotherapy or immunotherapy, along with enhanced nutritional support and inflammation control to improve outcomes. These patients would also benefit from more frequent follow-up visits to enable early detection of any changes in their condition. In contrast, patients with a high HALP score, who have a better prognosis, may require less frequent follow-ups, allowing for more efficient use of medical resources. However, our study has a few limitations. First, a larger sample size is required to further validate our results. Second, further exploration of the molecular mechanisms underlying these indicators is necessary to screen individuals at risk of a poor long-term prognosis.

## 5 Conclusion

In this study, we conclude that the HALP score could be utilized as an independent prognostic factor for patients with NSCLC. This information could assist clinicians in identifying high-risk groups with poor long-term prognoses and subsequently improve personalized treatment and optimized follow-up strategy.

## Data Availability

The original contributions presented in the study are included in the article/supplementary material. Further inquiries can be directed to the corresponding author.

## Footnotes

^1^ http://www.chictr.org.cn
